# The impact of large aneurysm diameter on the outcomes of thoracoabdominal aneurysm repair by fenestrated and branched endografts

**DOI:** 10.1093/ejcts/ezae387

**Published:** 2024-10-24

**Authors:** Enrico Gallitto, Nikolaos Tsilimparis, Paolo Spath, Gianluca Faggioli, Jan Stana, Antonino Logiacco, Carlota Fernandez-Prendes, Rodolfo Pini, Barbara Rantner, Chiara Mascoli, Antonio Cappiello, Mauro Gargiulo

**Affiliations:** Vascular Surgery, University of Bologna, DIMEC, Bologna, Italy; Vascular surgery unit, IRCCS Azienda Ospedaliero-universitaria di Bologna, Bologna, Italy; Department of Vascular Surgery, University Hospital, LMU Munich, Munich, Germany; Vascular Surgery, University of Bologna, DIMEC, Bologna, Italy; Vascular surgery unit, IRCCS Azienda Ospedaliero-universitaria di Bologna, Bologna, Italy; Vascular Surgery, University of Bologna, DIMEC, Bologna, Italy; Vascular surgery unit, IRCCS Azienda Ospedaliero-universitaria di Bologna, Bologna, Italy; Department of Vascular Surgery, University Hospital, LMU Munich, Munich, Germany; Vascular Surgery, University of Bologna, DIMEC, Bologna, Italy; Department of Vascular Surgery, University Hospital, LMU Munich, Munich, Germany; Vascular Surgery, University of Bologna, DIMEC, Bologna, Italy; Vascular surgery unit, IRCCS Azienda Ospedaliero-universitaria di Bologna, Bologna, Italy; Department of Vascular Surgery, University Hospital, LMU Munich, Munich, Germany; Vascular surgery unit, IRCCS Azienda Ospedaliero-universitaria di Bologna, Bologna, Italy; Vascular Surgery, University of Bologna, DIMEC, Bologna, Italy; Vascular Surgery, University of Bologna, DIMEC, Bologna, Italy; Vascular surgery unit, IRCCS Azienda Ospedaliero-universitaria di Bologna, Bologna, Italy

**Keywords:** Thoracoabdominal aortic aneurysm, Fenestrated/branched endovascular aneurysm repair, Large aortic diameter, Aneurysm diameter

## Abstract

**OBJECTIVES:**

Aim of the study was to analyse the impact of preoperative thoracoabdominal aneurysm diameter on the outcomes of fenestrated/branched endografting.

**METHODS:**

Patients who underwent endovascular thoracoabdominal repair at 2 European centres (2011–2021) were analysed. Median diameter was calculated; the third quartile was considered a cut-off. Outcomes were compared in 2 groups based on the diameter value. Primary endpoints were technical success, spinal cord ischaemia and 30-day/in-hospital mortality. Survival, freedom from reintervention and target visceral vessels instability were follow-up outcomes.

**RESULTS:**

Out of 247 thoracoabdominal aortic aneurysms, the median diameter was 65 mm, first quartile was 57 mm; third quartile was 80 mm, set as cut-off value. Fifty-nine (24%) patients had diameter ≥80 mm. Custom-made and off-the-shelf branched endograft were used in 160 (65%) and 87 (35%), respectively. Technical success was 93% (<80 mm: 91% vs ≥80 mm: 94%; *P* = 0.47). Twenty-three (9%) patients had spinal injury (<80 mm: 7% vs ≥80mm: 17%; *P* = 0.03). Twenty-two (9%) patients died within 30-day/in-hospital (<80 mm: 7% vs ≥80 mm: 15%; *P* = 0.06). Multivariate analysis did not report preoperative diameter ≥80 mm as significant risk factor for primary endpoints. The median follow-up was 13 (interquartile range: 2–37) months and at 3-year survival and freedom from reintervention rates were 65% and 62%, respectively. After univariate and multivariate analyses, preoperative diameter ≥80 mm was considered an independent risk factor for reinterventions [hazard ratio (HR): 1.9; 95% confidence interval (CI) 1.1–3.6; *P* = 0.04], and for target visceral vessels instability (HR: 3.1; 95% CI: 1.3–5.1; *P* = 0.04), occurred in 45 (18%) cases. However, after competing risk methods, preoperative diameter did not show significance for follow-up results.

**CONCLUSIONS:**

A preoperative thoracoabdominal aortic aneurysm diameter >80 mm has not had a direct impact on early technical and clinical outcomes. A diameter≥80 mm is considered risk factor for reinterventions and target vessels instability is considered separately during follow-up.

## GRAPHICAL ABSTRACT

**Figure:**
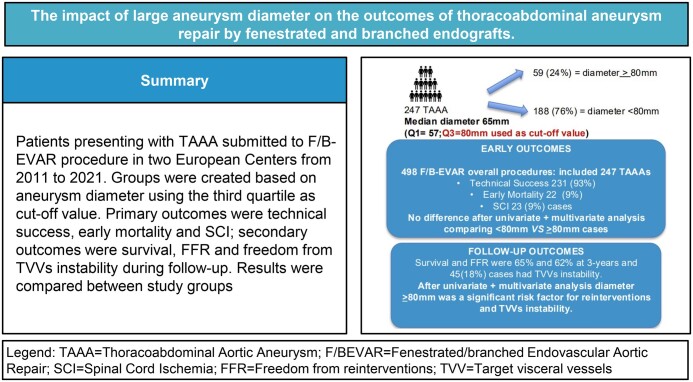

## INTRODUCTION

Fenestrated/branched endovascular aneurysm repair (F/B-EVAR) is an established technique to treat thoracoabdominal aneurysm (TAAAs) with reliable early and mid-term results in anatomically selected high-risk patients [1–3]. Several clinical/morphological predictors of technical and clinical failure have been proposed [4–6], but data about the role of preoperative TAAAs diameter are lacking.

In the presence of TAAAs with large diameter, F/B-EVAR technology could be associated with more technically demanding procedures. At the same time, some authors advocate a large aneurysm as a risk factor for rupture and requiring expedite repair [7, 8].

The aim of the study was to analyse the impact of the preoperative aneurysm diameter on the outcomes of TAAAs repairs by F/B-EVAR.

## METHODS

### Study design/patient selection

It was an observational, retrospective, multicentres study on consecutive patients that underwent endovascular TAAAs repair by custom-made/off-the-shelf F/B-EVAR (Cook platform, Bloomington, IN, US) at 2 European aortic centres, between 2011 and 2021 (Bologna: 2011–2021; Munich: 2018–2021). Patients treated by parallel graft, physician modified/in situ laser-fenestrations and hybrid repair were excluded.

### Endograft and procedure planning

Endograft planning was performed by a thoracoabdominal computed tomography angiography (CTA) and post-processing reconstructions on dedicated software (3Mensio—Vascular Imaging, Bilthoeven, Netherlands; Terarecon—Aquarius, Foster City, CA, USA). Target visceral vessel (TVVs) revascularization was obtained by fenestration or branch design according to diameter of aortic lumen at the level of TVVs and their orientations. Since 2012, off-the-shelf multibranched endografts (T-branch) were implanted in the presence of anatomical feasibility in patients with urgent TAAAs [7, 8, 10] or elective cases if the off-the-shelf solution did not lead to a longer proximal aortic coverage compared with a custom-made endograft [11].

Procedures, peri- and postoperative protocols have been accurately described in previous reports [1, 7, 12]. The specific spinal cord ischaemia (SCI) prevention protocols adopted were extensively described in previous papers published [7, 8, 12, 13] and patency of the left subclavian and at least one hypogastric artery was always guaranteed by surgical or endovascular means.

### Definitions and endpoints

The median preoperative aneurysm diameter was evaluated, and the median value was calculated. The third quartile Q3 was considered as a cut-off value in order to investigate the role of large diameter. Pre, intra and postoperative features/outcomes were defined and classified according with the current reporting standards [14].

Technical success (TS), postoperative SCI and 30-day/in-hospital mortality were assessed as primary outcomes. TS was defined as the ability to insert and deploy the main endograft and target vessels stent graft, with the exclusion of the aneurysm and patency of the TVV both at completion angiography and with extend by confirmatory CTA, in the absence of surgical conversion or mortality. Survival, freedom from reinterventions (FFR) and TVVs instability were assessed during follow-up, following the reporting standard definitions [14]. Patients were clustered according to the preoperative aneurysm diameter, with the diameter in the superior quartile over the Q3 cut-off value analysed as potential risk factor for the study’s outcomes.

### Follow-up

Before discharge, all patients underwent a thoracoabdominal CTA. The follow-up program was performed according to the protocol of each centre at 6, 12, 18, 24 months and yearly thereafter.

### Statistical analysis

Continuous data were reported by median and interquartile range (IQR Q1–Q3). Categorical data are expressed as frequency. Non-parametric Mann–Whitney *U*-test was used to investigate continuous variables differences. Fisher’s exact test was used for categorial univariate analysis, and survival and FFR were reported by Kaplan–Meier analysis. After univariate analysis, Logistic regression and Cox’s regression analysis were used for multivariate analysis the role of the variable in study for confounders, when significant in univariate analysis. Statistical analysis was performed by SPSS 25.0 for Windows software (SPSS, Inc., Chicago, IL, USA). Competing risk methods [15] were used for follow-up results using R software.

## RESULTS

### Patients

Out of 498 F/B-EVAR procedures, 247(50%) cases were TAAAs and they were considered in the study’s analysis. One hundred and eighty-three (74%) patients were male, median age was 73 (IQR: 67–78) years. The median preoperative TAAA diameter was 65 mm (IQR: 57–80 mm). Preoperative diameter 80 mm was considered as cut-off value for the subgroup analysis. Demographics and preoperative comorbidities are summarized in Table 1. Preoperative TAAA diameter was ≤80 mm in 188 (76%) and ≥80 mm in 59(24%) patients. Both subgroups resulted in comparable preoperative figures.

**Table 1:** Demographics, cardiovascular risk factors and preoperative comorbidities

	Overall		>80 mm	<80 mm	*P*-value
	*N*	%	*N* (%)	*N* (%)	<0.05
Total	247	100	59 (100)	188 (100)	
Male	183	74	46 (78)	137 (73)	0.43
Hypertension	230	93	55 (95)	175 (93)	0.63
Smoke					
Current	71	29	24 (41)	47 (25)	0.12
Previous	89	36	29 (49)	60 (32)	0.11
Dyslipidaemia	154	62	35 (60)	119 (63)	0.68
Diabetes	31	13	8 (14)	23 (12)	0.75
Chronic obstructive pulmonary disease	85	34	22 (38)	63 (33)	0.53
Coronary artery disease	72	29	14 (24)	58 (31)	0.32
Peripheral arterial occlusive disease	34	14	7 (12)	27 (14)	0.65
Atrial fibrillation	41	17	10 (17)	31 (17)	0.89
Anticoagulant oral therapy	24	10	5 (8)	19 (10)	0.76
Cerebrovascular disease	21	9	4 (7)	18 (9)	0.70
Body mass index ≥31	45	18	11 (18)	34 (18)	0.93
Chronic renal insufficiency	124	50	28 (48)	96 (51)	0.71
Dialysis	10	4	2 (3)	8 (4)	0.76
Previous aortic surgery	85	34	25 (42)	60 (32)	0.14
Open repair	77	31	16 (27)	61 (32)	0.44
Endovascular repair	74	30	19 (32)	55 (29)	0.66
Post-dissection TAAA	39	16	9 (15)	30 (16)	0.89
ASA III	117	48	26 (44)	91 (49)	0.76
ASA IV	128	52	33 (56)	97 (51)	0.65

	Median	IQR (Q1–Q3)			

Age (years)	73	67–78	75 (68–78)	70 (67–76)	0.370
Aneurysm diameter (mm)	65	57–80	92 (87–110)	60 (57–64)	<0.001

Procedures were performed in elective and urgent settings in 206 (83%) and 41 (17%) cases. Urgent repairs were more often performed for patients with diameter >80 mm (23 cases, 39%) when compared to patients with smaller aneurysm (18 cases 10%) (*P* < 0.001).

### Anatomical and endograft details

Eighty-five (34%) patients had a previous aortic repair, and 39 (16%) cases were chronic post-dissection TAAAs. Crawford’s extent I–III and IV were 154 (62%) and 93 (38%), respectively.

Proximal and distal sealing zones are summarized in Supplementary Material, Table S1. Supra-aortic trunk debranching and iliac branch devices were planned in 22 (9%) and 52 (9%) cases. Custom-made F/B-EVAR and off-the-shelf B-EVAR were used in 160 (65%) and 87 (35%), respectively. Details for TAAA’s extension and type of repair are listed in Supplementary Material, Table S2. Patients with larger diameter were more often treated with off-the-shelf B-EVAR (38 cases, 64%) than smaller diameter ones (49 cases, 26%) (*P* < 0.001). A multi-staged repair was planned in 151 (61%) cases.

### Procedure

All procedures were performed under general anaesthesia and CSF (cerebrospinalfluid) drainage was adopted in 192 (78%) cases. Surgical and percutaneous femoral access was performed in 143 (58%) and 104 (42%) cases, respectively. A surgical iliac conduit was performed in 23 (9%) cases to guarantee a safe endograft introduction. Surgical axillary access was performed in 182 (73%) cases.

Median total procedural and fluoroscopy time was 354 (IQR: 247–470) min and 124 (IQR: 86–606) min, respectively. Mean total radiation exposure (DAP) and iodinated contrast media administration were 435 223 (IQR: 5891–125 254) MGy/cm^2^ and 222 (IQR: 173–302) ml, respectively. Cases with preoperative TAAA diameter ≥80 mm did not show statistical differences when compared to smaller diameter ones.

TS was reported in 231(93%) cases. Failures are summarized in Supplementary Material, Table S3. In cases with preoperative TAAAs diameter <80 mm TS was in 177 (94%) patients versus diameter ≥80 mm in 54 (91%) cases (*P*=0.47).

### Early results

After procedure, all patients required intensive care unit with a mean recovery of 3 (IQR: 2–5) days. Cardiac and pulmonary morbidity was reported in 19 (8%) and 18 (7%) cases, respectively. Thirty-seven (15%) patients suffered renal function worsening and in 9 (4%) cases haemodialysis was necessary (4–2% permanent). Stroke and mesenteric ischaemic lesion occurred in 3 (1%) and 8 (3%) cases, respectively. Fifty-three (21%) patients underwent reinterventions within 30 postoperative days. Table 2 presents all the relevant primary and secondary endpoints and the univariate analysis between patients with preoperative diameter ≥80 mm versus smaller diameter. Twenty-three (9%) patients had SCI (Supplementary Material, Table S4) with 4 (2%) cases of Tarlov’s grade 0 at discharge. Perioperative diameter showed significance at univariate analysis (*P* = 0.03) but not after being adjusted for confounders (Table 3).

**Table 2:** Primary and secondary early clinical endpoints

	Overall		>80 mm	<80 mm	*P*-value
	*N*	%	*N* (%)	*N* (%)	<0.05
Total	247	100	59 (100)	188 (100)	
Primary outcomes					
Technical success	231	93	54 (91)	177 (94)	0.47
Spinal cord ischaemia	23	9	10 (17)	13 (7)	0.03
30-day mortality	22	9	9 (15)	13 (7)	0.06
Secondary outcomes					
Cardiac complications	19	8	7 (12)	12 (6)	0.15
Pulmonary complications	18	7	8 (14)	10 (5)	0.03
Renal function worsening	37	15	12 (20)	25 (13)	0.18
Postoperative dialysis	9	4	5 (8)	4 (2)	0.04
Stroke	3	1	1 (2)	2 (1)	0.13
Mesenteric ischaemia	8	3	2 (3)	6 (3)	0.72
Early reinterventions	53	21	18 (30)	35 (19)	0.06

**Table 3:** Univariate- and multivariate-adjusted analysis for risk factors associated with spinal cord ischaemia

	Univariate analysis	Multivariate logistic regression analysis		
	*P*-value	OR	95% CI	*P*-value
Preoperative TAAA diameter ≥80 mm	0.03	0.4	0.1–2.4	0.3
Preoperative risk factors				
Age over 73 years	0.48			
Hypertension	0.06			
Dyslipidaemia	0.30			
Diabetes	0.50			
Peripheral arterial occlusive disease	0.50			
Atrial fibrillation	0.20			
Previous aortic surgery	0.1			
Urgent cases	<0.001	3.2	1.2–8.6	0.01
Post-dissection TAAA	0.10			
Crawford’s extent I–III TAAAs	0.04	2.0	0.6–6.6	0.2
Intraoperative risk factors				
TAAA repair in single stage	0.30			
Debranching supra—aortic—trunks	0.40			
Off-the-shelf branched device	0.002	1.5	0.4–4.7	0.5
Custom-made device	0.40			
Aortic bi-iliac configuration	0.18			
Inverted limb	0.09			
Modified preloaded system for renal arteries	0.40			
Postoperative risk factors				
Postoperative cardiac morbidity	0.06			
Postoperative pulmonary morbidity	0.01	1.4	0.3–5.4	0.6
Postoperative renal morbidity	0.35			
Postoperative dialysis	0.2			
Stroke	0.3			
Mesenteric event	0.10			
Cerebral spinal fluid drainage	0.049			
Reinterventions @ 30-day	0.01	2.7	1.1–6.7	0.03

Results are reported if variables included show a *P*-value lower than 0.50 at univariate analysis.

CI: confidence interval; OR: odds ratio.

Median length of stay was 13 (IQR: 8–20) days, and 22 (9%) patients died within 30-day or during the hospitalization [elective: 11/206 (5%); urgent: 11/41 (27%)] and preoperative TAAAs diameter did not show significance (P:0.06).

### Follow-up results

**Figure 1:**
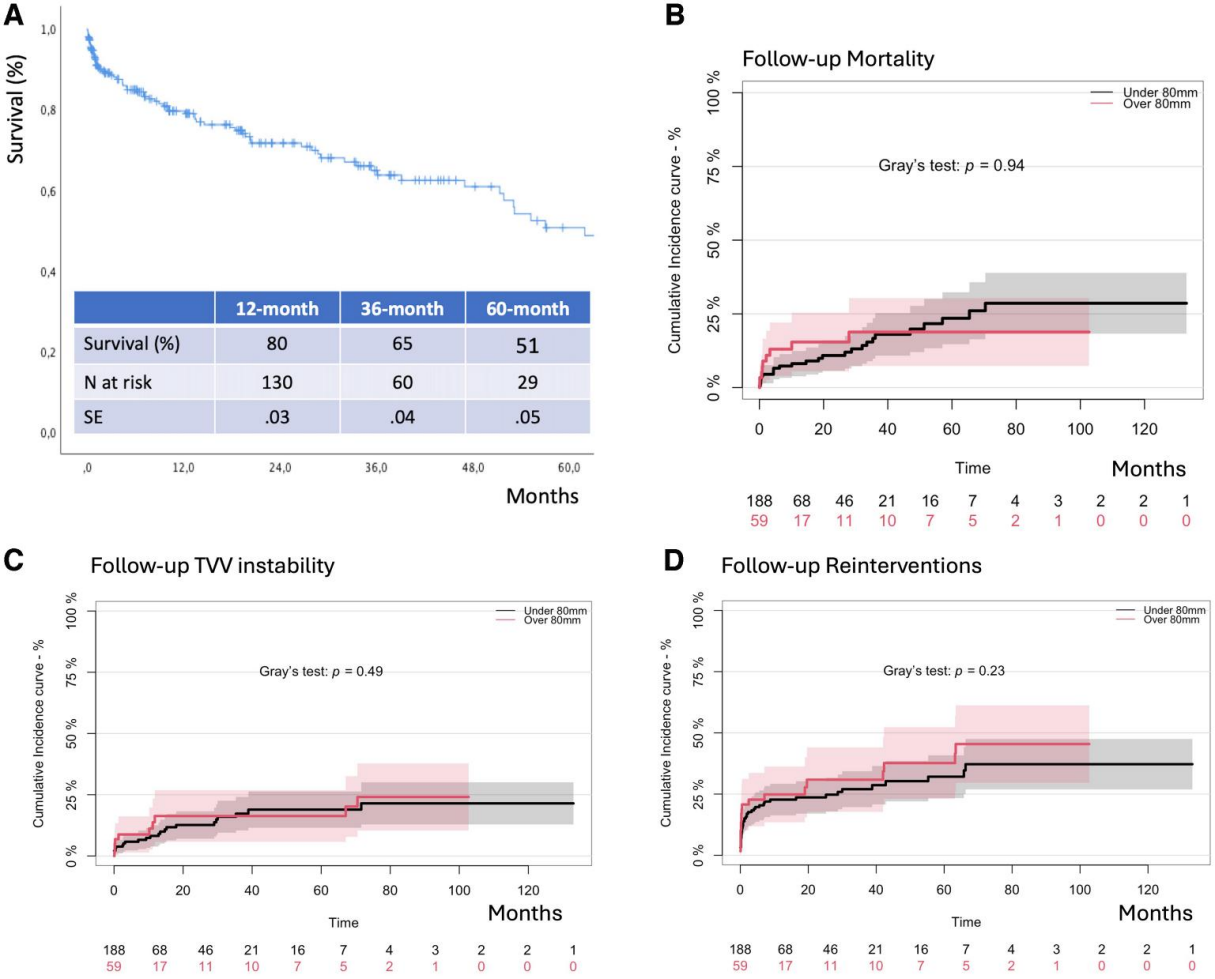
(**A**) Survival estimated by Kaplan–Meir analysis. (**B**–**D**) Follow-up regression analysis assessed by competing risk methods for mortality (**B**), target visceral vessels instability (**C**) and reinterventions (**D**) with impact of diameter over or under 80 mm assessed by Gray’s test.

The median follow-up was 13 (IQR: 2–37) months. No difference was found comparing large diameters versus small diameter patients (*P* = 0.63). Overall, 78 (32%) patients died (early + follow-up events) and causes of mortality were reported in Supplementary Material, Table S5. Survival was 65% at 3 years (Fig. 1A). At Kaplan–Meier (KM) analysis, preoperative TAAA diameter >80 mm versus <80 mm did not show statistical significance (*P* = 0.18).

Among the 225 patients who survived the 30-day/in-hospital mortality, TAAA diameter growth was detected in 27 (12%) patients during follow-up, while in 111 (49%) patients the sac remained stable and was reduced in 87 (39%) cases. No difference was found in sac expansion rates between large diameter (7/50–14%) versus small diameter (21/175–12%) patients (*P* = 0.76). Eighty-three (34%) patients underwent at least 1 reintervention (early + follow-up events), and in 27 (11%) cases, multiple reinterventions were necessary for an overall of 119 events; summarized in Supplementary Material, Table S6. FFR was 62% at 3 years with KM analysis. At KM analysis preoperative TAAA diameter >80 mm resulted in a significant factor (*P* = 0.02).

After Cox’s regression analysis, TAAA diameter ≥80 mm resulted in a relevant factor for reinterventions after adjustment for confounders (hazard ratio: 1.9; 95% confidence interval 1.1–3.6; *P* = 0.04) (Table 4).

**Table 4:** Univariate and multivariate Cox’s regression-adjusted analysis for risk factors associated with overall reinterventions (early + follow-up events)

	Univariate analysis	Cox’s regression multivariate analysis		
	*P*-value	HR	95% CI	*P*-value
Preoperative TAAA diameter ≥80 mm	0.02	1.9	1.1–3.6	0.04
Preoperative risk factors				
Age over 73 years	0.07			
Previous aortic surgery	0.30			
Crawford’s extent I–III TAAAs	0.001	2.8	1.5–5.1	0.001
Post-dissection TAAA	0.50			
TAAA repair in single stage	0.20			
Intraoperative risk factors				
Off-the-shelf branched device	0.05	1.2	0.6–2.2	0.5
Aortic bi-iliac configuration	0.40			
Abdominal tube configuration	0.40			
Inverted limb	0.10			
Modified preloaded system for renal arteries	0.50			
Technical success	0.19			
Postoperative risk factors				
Endoleak present at the discharge	0.50			
Postoperative aneurysm growth	0.002	3.6	1.6–8.4	0.002

Results are reported if variables included show a *P*-value lower than 0.50 at univariate analysis.

CI: confidence interval; HR: hazard ratio.

Overall, 45 (18%) patients had TVV instability, and univariate KM analysis reported preoperative TAAA diameter >80 mm as a significant factor (*P*=0.04). After Cox’s regression analysis, TAAA diameter ≥80 mm resulted in a relevant factor for TVVs instability after adjustment for confounders (Table 5).

**Table 5:** Univariate and multivariate Cox’s regression-adjusted analysis for risk factors associated with target visceral vessels instability

	Univariate analysis	Cox’s regression multivariate analysis		
	*P*-value	HR	95% CI	*P*-value
Preoperative TAAA diameter >80 mm	0.04	3.1	1.3–5.1	0.03
Preoperative risk factors				
Age over 73 years	0.06			
Urgent cases	0.06			
Previous aortic surgery	0.08			
Crawford’s extent I–III	0.03	1.2	0.2–2.1	0.90
Post-dissection TAAAs	0.03	3.3	1.2–3.2	0.03
Intraoperative risk factors				
TAAA repair in single stage	0.30			
Off-the-shelf branched device	0.30			
Custom-made device	0.40			
Aortic bi-iliac configuration	0.20			
Abdominal tube configuration	0.20			
Surgical iliac conduit	0.50			
Modified preloaded system for renal arteries	0.18			
Technical failure	0.001	2.7	1.1–3.1	0.005
Postoperative risk factors				
Endoleak present at the discharge	0.50			
Postoperative aneurysm growth	0.01	3.2	1.2–7.7	0.001

Results are reported if variables included show a *P*-value lower than 0.50 at univariate analysis.

CI: confidence interval; HR: hazard ratio.

Analysing competing events, cumulative incidence functions (Fig. 1B–D) showed that the probability for reinterventions and for TVV instability was not influenced by preoperative diameter.

## DISCUSSION

In this article, we report 247 consecutive TAAAs treated by F/B-EVAR in both elective and urgent settings with satisfactory results in terms of TS, spinal cord ischaemia, and 30-day/hospital mortality. Outcomes reported mid-term follow-up of survival, FFRs and with overall reduced rate of TVV instability and we investigated the impact of preoperative diameter larger than 80 mm on these outcomes.

Previous reports have proven several predictors of technical and clinical failure after F/B-EVAR in complex aortic aneurysms [4–6]. Data on infrarenal aneurysm diameter reported by Khashram *et al.* [16] suggest a lower follow-up survival in treated patients with preoperative larger diameter in both open and endovascular techniques. De Guerre *et al.* [17] reported that patients undergoing EVAR for abdominal aneurysm ≥65 mm had lower FFR and freedom from postoperative aneurysm rupture.

If we focus the attention on the TAAAs, data about the impact of preoperative diameter are currently lacking. In the present study, we have defined a preoperative large TAAA diameter considering a threshold ≥80 mm. The incidence of patients with large TAAA diameters was not negligible, representing about a quarter of the entire cohort.

As regards procedural outcomes, there was no difference in terms of TS based on preoperative diameter. Similar findings are reported for both 30-day/hospital mortality and SCI. According to the multivariable analysis, independent risk factors for SCI were urgent TAAA repair and need of reinterventions within 30-day. These factors should be carefully considered in high-risk patients, characterized by haemodynamic instability, impossibility to apply SCI prevention protocols and higher risk of postoperative respiratory and renal complications, as recently shown for non-elective and ruptured cases [7, 10, 18].

The median follow-up was 13 months, and it is comparable with the largest series of F/B-EVAR for TAAAs repair reported in the literature [1–3] and the rate of reinterventions is reported up to 20%, with about 1/3 of events defined as TVVs related [19].

When compared to open surgical repair, the result seems to favour the endovascular approach in the short period. When comparing these data overall early mortality was 9%, affected by the urgent repair. A recent meta-analysis over 17000 open elective TAAA patients reported a comparable overall mortality rates [20], while in case of urgency, the mortality rate of open surgery may overcome 30% [21]. Similarly, for SCI rates, the endovascular approach seems to confer a protective factor, compared to 12% reported after open repair [20]. Undoubtedly, rates of reinterventions are consistent with similar endovascular series [18] but higher when compared to open repair [20], testifying the need for close monitoring.

F/B-EVAR is a multi-modular device with multiple aortic and TVV components that can be considered potential localization of failure, causing high flow endoleaks or visceral vessel loss. These complications could be theoretically more frequent in extensive and large TAAAs. In a recent paper, published by Chait *et al.* [22], the distance between fenestration and ostium of the TVV (*gap distance*) was associated with TVV instability during follow-up. Moreover, in these situations, multiple segments are required and potential migrations may cause changes in bridging stents with subsequent risk of TVV instability. In the present experience, even if not investigated the precise diameter at the level of the paravisceral aorta, preoperative TAAA diameter ≥80 mm was an independent risk factor both for reinterventions and for TVVs instability when taking these outcomes alone. However, when calculating these factors by competing risk methods, the role of diameter seems to be not significant, even if higher crude % are shown for the large aneurysm group. These aspects suggest adapting peculiar technical attentions to create long/extensive overlap between aortic modules, bridging stentings and to plan longer sealing zone (≥3 cm) into TVVs in this specific subgroup of patients; however, a specific study should be performed to address specifically these outcomes.

The present study has several limits. First, it is a retrospective analysis using a single-brand platform. Results could be influenced by the retrospective analysis of events and changes, improvements of materials/technology during the study period. Second, it is a multicentre experience between centres with similar approaches and experience, thus results can be affected by different learning curve, hospital protocols and the use of both custom-made and off-the-shelf devices. Third, as expected, patients with larger aneurysm diameter underwent more often urgent repair due to the increased risk for rupture using an off-the-shelf branched device. Even if these aspects were analysed in multivariate-adjusted analysis, they may have influenced clinical outcomes. Eventually, outcomes are reported only at mid-term follow-up and important information about reinterventions and TVVs instability should be analysed considering dedicated designed studies considering the different results while analysing them as competing risks and the role of diameter that appeared to be not relevant when such refined analysis is then performed [15].

## CONCLUSION

TAAAs with large diameter can be endovascularly managed with satisfactory early technical and clinical outcomes as well as acceptable clinical results at mid-term follow-up. TAAAs with preoperative diameter ≥80 mm showed no difference in TS and perioperative mortality. TAAA diameter ≥80 mm is an independent risk factor for reinterventions and TVVs instability considered separately; however, after competing risk methods, preoperative diameter did not show significance. According to the present data, dedicated intra- and postoperative care should be required to reduce early and follow-up complications in patients with preoperative TAAA diameter ≥80 mm.

## Supplementary Material

ezae387_Supplementary_Data

## Data Availability

Data were collected in each centre from clinical records, shared anonymously and retrospectively analysed. All relevant data are within the manuscript and its Supporting Information files. The data of this study are available from the corresponding author upon reasonable request to the corresponding author DOI: 10.5281/zenodo.14013835. Due to its retrospective nature, individual informed consent was waived and Institutional Review Board was obtained in both centres (108/2022/Oss/AOUBo and LMU- 22–0483) in accordance with the Strengthening the Reporting of Observational Studies in Epidemiology (STROBE) guidelines for the observational studies [9].

## Notes

Presented at the Annual ESVS Meeting 2022, Rome, 20–23 September 2022. Fast track presentation and plenary session.

## ABBREVIATIONS

CTA: Computed tomography angiography
F/B-EVAR: Fenestrated/branched endovascular aneurysm repair
FFR: Freedom from reintervention
IQR: Interquartile range
SCI: Spinal cord ischaemia
TAAA: Thoracoabdominal aortic aneurysm
TS: Technical success
TVV: Target visceral vessel
