# Supplementary material for: The impact of large aneurysm diameter on the outcomes of thoracoabdominal aneurysm repair by fenestrated and branched endografts
Source: Eur J Cardiothorac Surg. 2024 Oct 24;66(5):ezae387. doi: 10.1093/ejcts/ezae387 (PMC11550190; doi:10.1093/ejcts/ezae387)
Supplement: ezae387_Supplementary_Data [file ezae387_supplementary_data.docx]

**Supplementary Table 1.** Proximal and distal aortic sealing zone according with the SVS reporting standards

| **Proximal sealing zone** | | |
| --- | --- | --- |
|  | **n** | **%** |
| 0 - 1 | 13 | 5 |
| 2 | 16 | 7 |
| 3 | 73 | 30 |
| 4 | 53 | 21 |
| 5 | 92 | 37 |
| Overall | 247 | 100 |
| **Distal sealing zone** | | |
|  | **n** | **%** |
| 9 | 80 | 32 |
| 10 - 11 | 167 | 68 |
| Overall | 247 | 100 |
| Right iliac axis - 167 |  |  |
| 10 | 146 | 87 |
| 11 | 21 | 13 |
| Left iliac axis - 167 |  |  |
| 10 | 145 | 87 |
| 11 | 22 | 13 |

**Supplementary Table 2.** Thoracoabdominal aneurysm extension and details of diameter and repair.

| **Thoracoabdominal Crawford’s Extension** |  | **TAAA diameter** | | **>80mm** | **t-branch device** | **CMD** |
| --- | --- | --- | --- | --- | --- | --- |
|  | **n (%)** | **Median** | **n (%)** | | **n (%)** | **n (%)** |
| I | 29 (12) | 69 | 7 (12) | | 10 (11) | 19 (12) |
| II | 67(27) | 71 | 25 (42) | | 37 (43) | 30 (19) |
| III | 58 (23) | 67 | 9 (15) | | 22 (25) | 36 (22) |
| IV | 93 (38) | 64 | 18 (31) | | 18 (21) | 75 (47) |
|  |  |  |  | |  |  |
| Overall | 247 (100) | 65 | 59 (100) | | 87 (100) | 160 (100) |

**Suppementary Table 3.** Technical failures in both elective and urgent cases: incidence and causes.

|  | **n** | **%** |
| --- | --- | --- |
| *Overall* | 16 / 247 | 7.0 |
| Target visceral vessels loss  Impossibility to cannulate renal artery  Superior mesenteric artery stentgraft thrombosis | 7 / 247  6  1 | 3.0 |
| Target visceral vessels related endoleak | 5 / 247 | 2.0 |
| Target visceral vessels unrelated endoleak (I-III) | 1 / 247 | 0.5 |
| Renal bleeding | 1 / 247 | 0.5 |
| Intraoperative death | 2 / 247 | 1.0 |
|  |  |  |
| *Elective* | 12 / 206 | 6.0 |
| Target visceral vessels loss  Impossibility to cannulate renal artery  Superior mesenteric artery stentgraft thrombosis | 7 / 206  6  1 | 3.5 |
| Target visceral vessels related endoleak | 5 / 206 | 2.5 |
|  |  |  |
| *Urgent* | 4 / 41 | 10 |
| Intraoperative death | 2 / 41 | 5 |
| Renal bleeding | 1 / 41 | 2.5 |
| Endoleak type III (between TEVAR modules) | 1 / 41 | 2.5 |

**Supplementary Table 4.** Cases of spinal cord ischemia. Anatomical and clinical details for each event

| **N** | **Clinical setting** | **Crawford’s extent** | **Stages of repair** | **CSF drainage** | **Previous aortic surgery** | **TAAA diameter**  (mm) | **SCI grade T0** | **SCI grade T1** |
| --- | --- | --- | --- | --- | --- | --- | --- | --- |
| 1 | Elective | I-III | Single | Yes | No | 70 | 5 | 0 |
| 2 | Elective | I-III | Multiple | Yes | No | 80 | 3 | 4 |
| 3 | Elective | I-III | Multiple | Yes | No | 60 | 3 | 0 |
| 4 | Elective | I-III | Single | Yes | No | 80 | 2 | 0 |
| 5 | Urgent | I-III | Single | No | Yes | 65 | 0 | 0 |
| 6 | Elective | IV | Single | No | No | 65 | 0 | 0 |
| 7 | Urgent | I-III | Single | No | Yes | 70 | 3 | 4 |
| 8 | Urgent | I-III | Single | Yes | No | 100 | 3 | 3 |
| 9 | Elective | IV | Multiple | No | Yes | 120 | 4 | 5 |
| 10 | Elective | I-III | Multiple | Yes | No | 73 | 4 | 4 |
| 11 | Urgent | I-III | Multiple | Yes | No | 85 | 3 | 4 |
| 12 | Urgent | I-III | Single | Yes | No | 110 | 1 | 0 |
| 13 | Urgent | IV | Single | No | Yes | 100 | 3 | 4 |
| 14 | Elective | I-III | Multiple | Yes | No | 68 | 0 | 0 |
| 15 | Elective | I-III | Multiple | Yes | No | 80 | 5 | 5 |
| 16 | Elective | I-III | Multiple | Yes | No | 40 | 5 | 4 |
| 17 | Elective | I-III | Multiple | Yes | No | 70 | 0 | 0 |
| 18 | Urgent | I-III | Single | Yes | No | 100 | 4 | 1 |
| 19 | Elective | I-III | Multiple | Yes | No | 60 | 5 | 3 |
| 20 | Urgent | I-III | Single | Yes | No | 70 | 3 | 5 |
| 21 | Urgent | I-III | Single | Yes | No | 115 | 5 | 3 |
| 22 | Elective | IV | Multiple | Yes | No | 64 | 3 | 0 |
| 23 | Urgent | I-III | Multiple | Yes | No | 53 | 3 | 5 |

CSF: cerebral spinal fluid; TAAA: thoracoabdominal aortic aneurysm; SCI: spinal cord ischemia.

**Supplementary Table 5.** Causes and timing of overall mortality

|  | **Within 30-day**  N - % | **After 30-day**  N - % | **Overall**  N - % |
| --- | --- | --- | --- |
| Aortic - rupture | 1 | 1 | 2 |
| Cancer | 0 | 6 | 6 |
| Cardiac | 3 | 13 | 16 |
| Cerebral hemorrhage | 2 | 1 | 3 |
| Endograft Infection | 0 | 2 | 2 |
| Gastroenterological | 1 | 1 | 2 |
| Multi organ failure | 3 | 4 | 7 |
| Pulmonary embolism | 1 | 1 | 2 |
| Respiratory | 3 | 2 | 5 |
| Renal | 1 | 0 | 1 |
| Senectus | 0 | 10 | 10 |
| Sepsis | 0 | 2 | 2 |
| Stroke | 0 | 1 | 1 |
| Unknow | 0 | 19 | 19 |
| Overall | 15 - 19 | 63 - 81 | 78 - 100 |

**Supplementary Table 6.** Reinterventions

|  | **n** | **%** |
| --- | --- | --- |
| Patients with reinterventions | 83 / 247 | 34 |
| Patients with reinterventions within 30-day | 53 / 83 | 64 |
| Patients with reinterventions after 30-day | 30 / 83 | 36 |
| Patients with multiple reinterventions | 27 / 83 | 33 |
| Overall reinterventions | 119 /119 | 100 |
| Access related | 48 /119 | 40 |
| Aortic - iliac related | 25 /119 | 21 |
| Target Visceral vessel related | 46 /119 | 39 |

**STROBE Statement—checklist of items that should be included in reports of observational studies
Study title: The impact of large aneurysm diameter on the outcomes of thoracoabdominal aneurysm repair by fenestrated and branched endografts.**

|  | Item No. | Recommendation | Page  No. | Relevant text from manuscript |
| --- | --- | --- | --- | --- |
| **Title and abstract** | 1 | (*a*) Indicate the study’s design with a commonly used term in the title or the abstract | 2 |  |
|  |  | (*b*) Provide in the abstract an informative and balanced summary of what was done and what was found | 3 |  |
| Introduction | | | |  |
| Background/rationale | 2 | Explain the scientific background and rationale for the investigation being reported | 5 |  |
| Objectives | 3 | State specific objectives, including any prespecified hypotheses | 5 |  |
| Methods | | | |  |
| Study design | 4 | Present key elements of study design early in the paper | 6 |  |
| Setting | 5 | Describe the setting, locations, and relevant dates, including periods of recruitment, exposure, follow-up, and data collection | 6 |  |
| Participants | 6 | (*a*) *Case-control study*—Give the eligibility criteria, and the sources and methods of case ascertainment and control selection. Give the rationale for the choice of cases and controls | 7 | ***Definitions and endpoints*** |
|  |  | (*b*) *Cohort study*—For matched studies, give matching criteria and number of exposed and unexposed  *Case-control study*—For matched studies, give matching criteria and the number of controls per case | - | All comers study of consecutive patients. |
| Variables | 7 | Clearly define all outcomes, exposures, predictors, potential confounders, and effect modifiers. Give diagnostic criteria, if applicable | 7 |  |
| Data sources/ measurement | 8* | For each variable of interest, give sources of data and details of methods of assessment (measurement). Describe comparability of assessment methods if there is more than one group | *7* |  |
| Bias | 9 | Describe any efforts to address potential sources of bias | 8 | Statistical analysis section |
| Study size | 10 | Explain how the study size was arrived at | - | All comers study of consecutive patients. |

| Quantitative variables | 11 | Explain how quantitative variables were handled in the analyses. If applicable, describe which groupings were chosen and why | 8 |  |
| --- | --- | --- | --- | --- |
| Statistical methods | 12 | (*a*) Describe all statistical methods, including those used to control for confounding | 8 |  |
|  |  | (*b*) Describe any methods used to examine subgroups and interactions | 8 |  |
|  |  | (*c*) Explain how missing data were addressed | - |  |
|  |  | (*d*) *Cohort study*—If applicable, explain how loss to follow-up was addressed  *Case-control study*—If applicable, explain how matching of cases and controls was addressed  *Cross-sectional study*—If applicable, describe analytical methods taking account of sampling strategy | - |  |
|  |  | (*e*) Describe any sensitivity analyses | - |  |
| Results | | | | |
| Participants | 13* | (a) Report numbers of individuals at each stage of study—eg numbers potentially eligible, examined for eligibility, confirmed eligible, included in the study, completing follow-up, and analysed | 8 |  |
|  |  | (b) Give reasons for non-participation at each stage | 8 |  |
|  |  | (c) Consider use of a flow diagram | - | Not applied since all comers TAAA aneurysm were involved (summarized shortly in central image) |
| Descriptive data | 14* | (a) Give characteristics of study participants (eg demographic, clinical, social) and information on exposures and potential confounders | 8 |  |
|  |  | (b) Indicate number of participants with missing data for each variable of interest | - |  |
|  |  | (c) *Cohort study*—Summarise follow-up time (eg, average and total amount) | 10 |  |
| Outcome data | 15* | *Cohort study*—Report numbers of outcome events or summary measures over time | *-* |  |
|  |  | *Case-control study—*Report numbers in each exposure category, or summary measures of exposure | *9-10* | *Tables 1-2* |
|  |  | *Cross-sectional study—*Report numbers of outcome events or summary measures | *-* |  |
| Main results | 16 | (*a*) Give unadjusted estimates and, if applicable, confounder-adjusted estimates and their precision (eg, 95% confidence interval). Make clear which confounders were adjusted for and why they were included | 10-11 | Tables 2-3-4-5 |
|  |  | (*b*) Report category boundaries when continuous variables were categorized | - |  |
|  |  | (*c*) If relevant, consider translating estimates of relative risk into absolute risk for a meaningful time period | - |  |

| Other analyses | 17 | Report other analyses done—eg analyses of subgroups and interactions, and sensitivity analyses | - |  |
| --- | --- | --- | --- | --- |
| Discussion | | | | |
| Key results | 18 | Summarise key results with reference to study objectives | 11-12 |  |
| Limitations | 19 | Discuss limitations of the study, taking into account sources of potential bias or imprecision. Discuss both direction and magnitude of any potential bias | 14 |  |
| Interpretation | 20 | Give a cautious overall interpretation of results considering objectives, limitations, multiplicity of analyses, results from similar studies, and other relevant evidence | 13-14 |  |
| Generalisability | 21 | Discuss the generalisability (external validity) of the study results | 15 |  |
| Other information | |  | | |
| Funding | 22 | Give the source of funding and the role of the funders for the present study and, if applicable, for the original study on which the present article is based | 15 |  |

*Give information separately for cases and controls in case-control studies and, if applicable, for exposed and unexposed groups in cohort and cross-sectional studies.

**Note:** An Explanation and Elaboration article discusses each checklist item and gives methodological background and published examples of transparent reporting. The STROBE checklist is best used in conjunction with this article (freely available on the Web sites of PLoS Medicine at http://www.plosmedicine.org/, Annals of Internal Medicine at http://www.annals.org/, and Epidemiology at http://www.epidem.com/). Information on the STROBE Initiative is available at www.strobe-statement.org.
